# Comfort evaluation of ZnO coated fabrics by artificial neural network assisted with golden eagle optimizer model

**DOI:** 10.1038/s41598-022-10406-6

**Published:** 2022-04-15

**Authors:** Nesrine Amor, Muhammad Tayyab Noman, Michal Petru, Neethu Sebastian

**Affiliations:** 1 Technical University of Liberec, 461 17 Studentská 1402/2, Liberec 1, Czech Republic; 2 Institute of Organic and Polymeric Materials, National Taipei University of Technology, No. 1, Section 3, Zhongxiao East Road, Taipei, 106 Taiwan, ROC

**Keywords:** Nanoparticles, Computational methods, Nanoparticles, Synthesis and processing, Materials science, Nanoscience and technology

## Abstract

This paper introduces a novel technique to evaluate comfort properties of zinc oxide nanoparticles (ZnO NPs) coated woven fabrics. The proposed technique combines artificial neural network (ANN) and golden eagle optimizer (GEO) to ameliorate the training process of ANN. Neural networks are state-of-the-art machine learning models used for optimal state prediction of complex problems. Recent studies showed that the use of metaheuristic algorithms improve the prediction accuracy of ANN. GEO is the most advanced methaheurstic algorithm inspired by golden eagles and their intelligence for hunting by tuning their speed according to spiral trajectory. From application point of view, this study is a very first attempt where GEO is applied along with ANN to improve the training process of ANN for any textiles and composites application. Furthermore, the proposed algorithm ANN with GEO (ANN-GEO) was applied to map out the complex input-output conditions for optimal results. Coated amount of ZnO NPs, fabric mass and fabric thickness were selected as input variables and comfort properties were evaluated as output results. The obtained results reveal that ANN-GEO model provides high performance accuracy than standard ANN model, ANN models trained with latest metaheuristic algorithms including particle swarm optimizer and crow search optimizer, and conventional multiple linear regression.

## Introduction

Zinc oxide (ZnO) is an inorganic compound used in various products and applications including food supplements, cosmetics, plastics, textiles, ceramics, paints, batteries and many more. ZnO in nanoforms is available in different dimensions and morphologies including nanoparticles, nanowires, nanosheets and nanoflowers. ZnO nanoparticles (ZnO NPs) are widely used in photocatalysis, self-cleaning and antimicrobial applications^1–3^. The use of ZnO for thermophysiological and sensorial comfort is also significant from different aspects. Thermophysiological properties are influential parameters that play important role in the evaluation of fabric comfort^4^. In a recent study, Noman *et al.* synthesized and coated ZnO NPs on woven textiles by sonication and practically evaluated thermal resistance, heat flow, thermal diffusivity, accumulative One-way transport index and wetting time^5^. In this extended study, a prediction model is designed based on the application of a latest machine learning algorithm (GEO) and its synchronization with ANN in order to improve the training process of ANN. The benefit of using ANN in this work, is the adaptation of existing relationship without any physical mechanism. The resulted ANN-GEO model works in three ways i.e., correlates the actual response with the process variables, analyses the predicted response of each variable and indicates the better approach.

ANN is an efficient machine learning tool suitable for the prediction of output response when input conditions are not defined^6–8^. Khude *et al.* used ANN with adaptive network-based fuzzy inference system (ANFIS) for antimicrobial evaluation of knitted fabrics. The results reveal that ANFIS performed better under small number of data sets^9^. Knanat *et al.* applied standard ANN for the evaluation of thermal resistance of knitted fabrics. Two different models of ANN were developed based on input conditions. The results of both models showed excellent prediction of thermal resistance^10^. Lu *et al.* combined ANN with multiple linear regression (MLR) for tensile strength of wool fibers. A high correlation was observed between actual and predicted values. However, ANN showed higher accuracy and lower error than MLR^11^. In a study, Malik *et al.* applied ANN for comfort evaluation of woven fabrics. ANN was trained with feedforward back propagation composed of Bayesian regularization and Levenberg-Marquardt functions. The results showed that ANN adjusts the data sets with lower mean absolute error (MAE)^12^. In another work, Malik *et al.* used ANN for loom parameters and fabric properties including porosity, pore size and air permeability. The obtained results showed that ANN is an excellent prediction tool with minimum error^13^. Wong *et al.* combined ANN with fuzzy logic (FL) for clothing comfort and results reveal better correlation of input and out conditions^14^. Mishra applied ANN to predict yarn strength. Selected variables were yarn count, yarn crimps and yarn strengths in both longitudinal and transverse directions. The results reveal a percentage increase in both directions^15^.

In an experimental study, El-Geiheini *et al.* investigated yarn types and applied ANN for yarn tenacity and elongation. The results showed ANN is suitable for the evaluation of various properties with minimum error^16^. In another study, Erbil *et al.* applied ANN and regression for tensile strength of rotor yarns. ANN was trained with Levenberg-Marquardt backpropagation. In addition, a comparison of ANN and regression was performed for prediction efficiency. The results demonstrated that ANN gives better prediction output than regression^17^. Breuer *et al.* applied ANN for short fiber composites under representative volume element database. The elastic properties of short fiber reinforced plastics were evaluated and results showed that ANN predicts the stiffness in good manner^18^. Wang *et al.* used ANN to predict the tensile strength of ultrafine glass fiber felts. The results are modelled with mean diameter, bulk density and resin content. ANN simulation showed high prediction accuracy with low mean relative errors^19^. Farook *et al.* used ANN for cotton fibre maturity. The results showed low error for ANN^20^. Unal *et al.* used ANN and regression for single jersey knitted fabrics to predict air permeability and bursting strength of knit structures. Simulation results showed that both methods were good for prediction^21^. Farooq *et al.* applied ANN for shade change prediction of dyed knitted fabrics. The observed results showed that ANN provides high prediction accuracy for shade change^22^. Recently, Amor *et al.* applied ANN and MLR on functional properties of nano $\hbox {TiO}_2$ coated composites. Simulation results showed that for prediction accuracy, ANN outperformed MLR^23^. In another study, Amor *et al.* used deep neural network (DNN) for the prediction of methylene blue removal. DNN model showed better results than MLR^24^. The literature discussed above reveal that multilayer perceptron (MLP) is a popular class of feedforward ANN model, and extensively used ANN algorithm in textiles and composites industries^25^.

The application of metaheuristic algorithms (particle swarm optimizer, genetic algorithm, crow search optimizer) for accuracy improvement of ANN has gained considerable attention. Genetic algorithm (GA) was used with ANN to improve pilling performance^26^, yarn tenacity^27^, ultraviolet protection factor (UPF)^28^. Grey wolf optimizer (GWO) was combined with ANN to detect and predict the coating thickness by hyperspectral images^29^. ANN trained with particle swarm optimization (PSO) used for thermal properties^30^ and trained with crow search algorithm (CSA) used for the prediction of functional properties of nanocomposites^31^. Recently, a novel nature-inspired metaheuristic algorithm known as GEO, has been introduced to solve global optimization. GEO is inspired by the intelligence of golden eagles for hunting by tuning their speed according to the spiral trajectory. GEO is distinguishable from GA, PSO, CSA and dragonfly algorithm (DA) by its setting parameters that make the process more intuitive and effective in solving complex problems^32^. In addition, the application of GEO in real-world applications showed its potential and suggested its use in other fields especially textiles and polymer composites.

This study provides the following benefits: (1) Proposing the use of GEO to train MLP. (2) Investigating the accuracy of propose ANN-GEO for the prediction of comfort properties of ZnO coated fabrics by creating a relationship between ZnO, comfort properties and ultrasonic irradiations. The amount of ZnO NPs, fabric mass and fabric thickness were selected as input variables and comfort properties i.e., thermal diffusivity, thermal resistance, heat flow, accumulative One-way transport index and wetting time were considered as output responses. (3) Comparing the performance of ANN-GEO with standard ANN, standard MLR and ANN trained by PSO, CSA and GA algorithms.

## Material and methods

### Materials

Two types of plain weave woven fabric ($100\%$ cotton and polyester) were used for samples preparation. Chemicals i.e., sodium hydroxide, zinc chloride and ethanol were received from sigma aldrich. The variables selected for this study were ZnO NPs coated amount, fabric mass measured as gram per square meter (GSM) and fabric thickness before and after treatment. The combination of these variables is described in Table 1.

**Table 1:** The input variables for experimental design.

Sample	ZnO NPs coated amount [*ppm*]	GSM $[g m^{2}]$	Thickness [*mm*]
1	–	110	0.25
2	581	115	0.31
3	1090	118	0.38
4	–	224	0.66
5	598	229	0.72
6	1110	233	0.77
7	–	118	0.32
8	493	124	0.36
9	1032	128	0.41
10	–	230	0.66
11	583	234	0.78
12	1096	238	0.84

### Conditioning

Before any treatment, all the samples were conditioned at temperature i.e., ${23\pm 2}$ $^\circ C$ and relative humidity i.e., ${65\pm 2}$ for one day according to ASTM standard D 1776-16. After that, fabric mass was calculated by ASTM D 3776. ASTM D 1777-96 (2019) standard was used to measure fabric thickness before and after the application of ZnO NPs.

### Application of ZnO NPs

Synthesis and coating of ZnO NPs were performed on all the samples according to the procedure as reported in our previous studies^33,34^. Fabric samples were individually immersed in water and then different amount of $\hbox {ZnCl}_2$ was added. Later on, NaOH in granular form was added. In order to complete the reaction, sonication was applied for 1 h. After sonication, samples were squeezed on padder and dried in an oven. ZnO NPs were characterised for their morphology and topography. Ultrahigh resolution scanning electron microscopy (UHR-SEM) was used to characterise the morphological and topographical changes. ZnO NPs coated amount was calculated by inductively coupled plasma atomic emission spectroscopy (ICP-AES).

### Artificial neural network

Prediction of thermophysiological properties of ZnO NPs coated samples is a challenging task due to the complex relationship between applied chemicals and obtained results. ANN has achieved important milestones in the field of artificial intelligence (AI)^35^. ANN models are inspired by biological neural networks that allow them to capture linear or nonlinear complex relationships between dependent and independent variables^36^. It includes large groups of neurons connected by axons. The artificial neuron has multiple inputs that are weighted, summed up and followed by an activation function or transfer function. Every neuron receives input from various sources and applies activation function to provide the desired results. The advantages of ANN are exploration, creation and derivation of new data through training process^37^.

MLP is a feedforward ANN model in which one direction processes the information from input to output neurons under multiple hidden layers. MLP deals with non-linear models by decreasing the targeted error by tailoring weight and biases^38^. In MLP, training process is implemented in four steps: 1) Initialization: assuming that there is no prior information available and initializing the weights and thresholds values. 2) Forward propagation: the inputs of ANN model are the experimental data and their effects propagate at different stages by moving forward the network layer by layer which creates the network output. 3) Error computation: the error vector is calculated by actual and predicted output difference. 4) Backward propagation: the calculated error vector propagates backwards and synaptic weights are adjusted.

**Figure 1:**
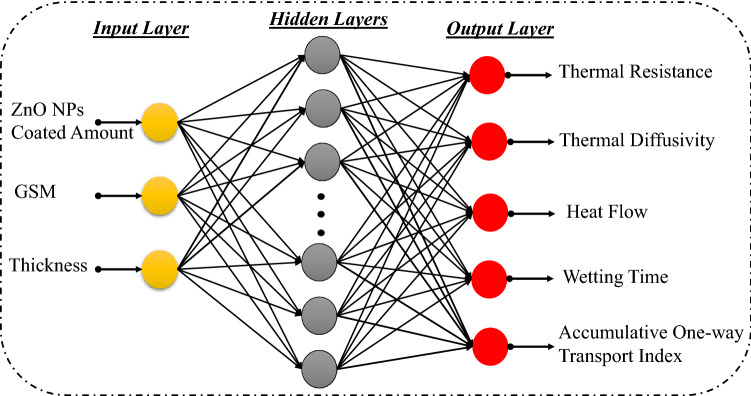
ANN model for the prediction of comfort properties of ZnO coated fabrics.

Generally, ANN is used to predict the output variables $y= [y_{1}, \cdots , y_{m}]$ for a given set of input variables $x= [x_{1}, \cdots , x_{k}]$ from their training values. The results depend on weights $w= [w_{1}, \cdots , w_{k}]$. The relationship between input and output of ANN model is presented in the following equation^39,40^:

1 $$\begin{aligned} y =\varphi \left( \sum _{i} w_i *x_i +b \right) \end{aligned}$$

where, *y* represents the desired output. $x_i$ represents the selected $i^{th}$ input. $w_i$ represents the $i^{th}$ weight and *b* is the bias. $\varphi$ represents the activation function. More theoretical detail of ANN models with their training algorithms is provided by various researchers^41–45^. In the ANN model, by increasing the number of network layers, the results will be significantly more accurate. However, this increase will make the training process more difficult to fit and will lead to a time-consuming process. We, therefore, adopted the classical structure of feedforward ANN (MLP model) in the present work. The classical structure of feedforward ANN consists of three layers (one input layer, one output layer and one hidden layer). Figure 1 illustrates the schematic ANN model for this study.

The recommended quantity to train the network, is from $60\%$ up to $90\%$ of the samples^46^. The datasets of Table 1 were divided into three parts (training, validation and testing) for the proposed ANN model, where $60\%$ of total data was used to train the network, $15\%$ was used for validation and remaining $25\%$ was used for testing. Once the training process is completed, the developed model is validated for unseen data during training. Random sub-sampling cross-validation method was applied to evaluate the topology and training of proposed model. The training inputs vectors are shown in Table 1. The output vectors include thermal resistance, thermal diffusivity, heat flow, accumulative One-way transport index and wetting time. The selected number of input and output nodes were 3 and 5 respectively.

### ANN optimized with golden eagle optimizer

- **Golden eagle optimizer**

GEO is a new metaheuristic method that was introduced very recently to solve global optimization problems. GEO algorithm is inspired and mathematically modeled by the intelligence of the golden eagles based on controlling the speed of their spiral track. Golden eagle is a special kind of swarm that has a greater propensity to cruise around and search for prey at the start of hunt. By controlling these two components, i.e., cruise propensity and attack propensity, GEO is quickly able to hunt the best available prey in the feasible area.

The golden eagle in cruising and hunting has a unique feature i.e., occurs in a spiral trajectory which means that prey is generally on one side of the eagle. This enables them to control target prey carefully and boulders to find a suitable angle of attack. At the same time, they check other areas for better food. The hunting method of golden eagles mainly depend on the following feature: they have an intelligent memory that allows them to memorize the propensity for both cruise and attack during the flight.

**Figure 2:**
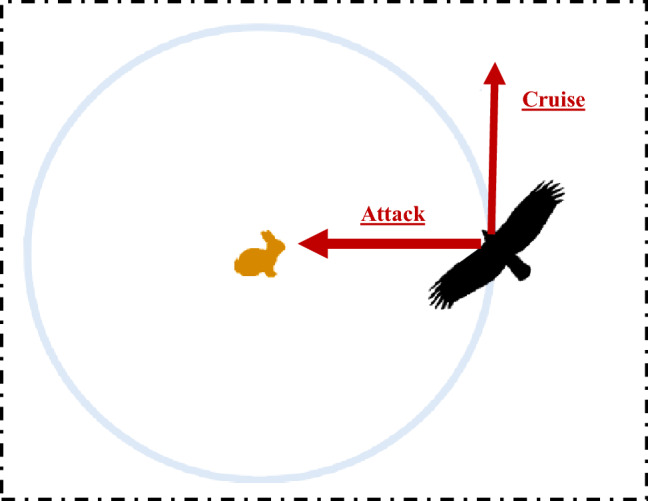
Spiral movement of golden eagles^32^.

**Figure 3:**
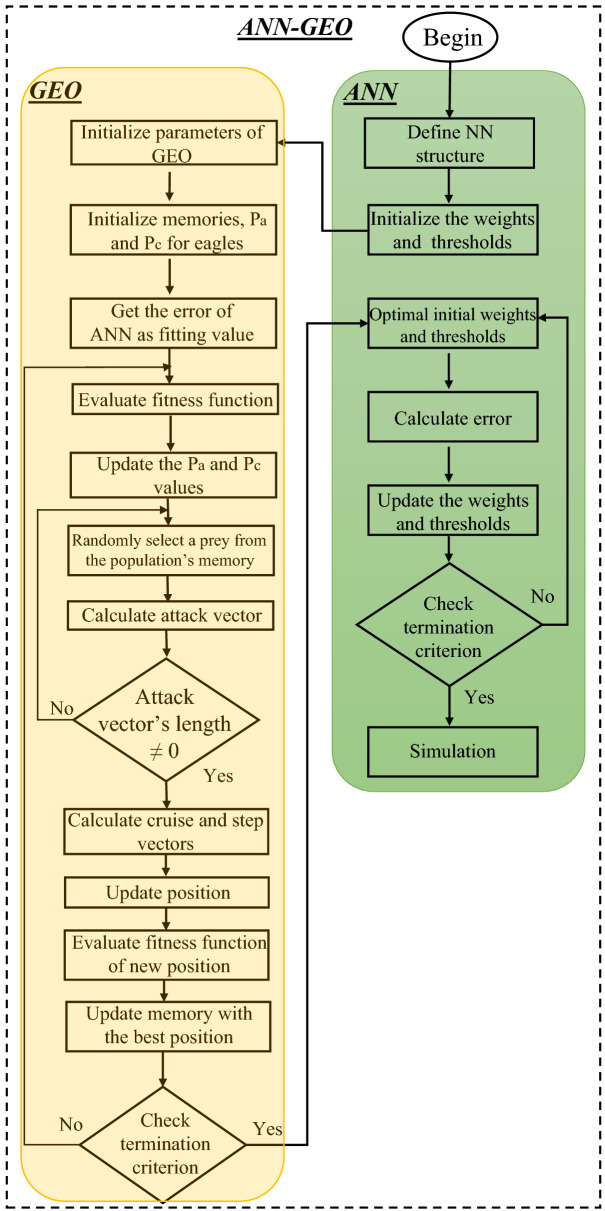
ANN-GEO model for the prediction of thermophysiological comfort properties of ZnO NPs coated fabrics.

The mathematical formulations of golden eagles to mimic the movements for searching the prey are mainly described by:

- **The spiral movement of golden eagles:** In GEO, every golden eagle keeps in its memory the best visited position so far. The eagle has an attraction towards the cruise and towards attacking the prey simultaneously to search for better food. Figure 2 depicts the cruise and attack vectors in 2*D* space. At every iteration, every golden eagle *j* can randomly chooses a prey that has been caught by another golden eagle *l* and circles around the best position visited by golden eagle *l* so far. The golden eagle *j* also has the feature of selection to circle its own memory; thus, we have $l \in \{1, 2, \cdots , N_{GE}\}$, where $N_{GE}$ represents the number of golden eagles.
- **Prey selection:** At every iteration, every golden eagle should select a prey to carry out the cruise and attack operations. In addition, each golden eagle chooses the desired prey from the memory of the whole flock. Therefore, the cruise and attack vectors are computed according to the selected prey. After that, it checks its memory If the new location is better than the previous location, then the memory is updated with the new finding.
- **Attack:** The attack can be described using a vector starting from the actual position of golden eagle *j* and ending in the position of the prey in the eagle’s memory, as follows:
  2 $$\begin{aligned} \overrightarrow{\mathrm{A}_{\mathrm{j}}}= \overrightarrow{\mathrm{X}_{\mathrm{l}}^{*}}-\overrightarrow{\mathrm{X}_{\mathrm{j}}}, \end{aligned}$$
  where $\overrightarrow{\mathrm{A}_{\mathrm{j}}}$ represents the attack vector of golden eagle *j*, $\overrightarrow{\mathrm{X}_{\mathrm{l}}^{*}}$ represents the best position visited by eagle *l* so far, and $\overrightarrow{\mathrm{X}_{\mathrm{j}}}$ represents the current position of eagle *j*.
- **Cruise:** The cruise vector is a perpendicular vector to the attack vector and tangent to the circle. It is also known as linear speed of golden eagle to attack the prey. The destination point on the cruise vector is given below:
  3 $$\begin{aligned} \overrightarrow{\mathrm{C}_{\mathrm{j}}}= \frac{d-\sum _{f,f \ne j} a_f}{a_j}, \end{aligned}$$
  where, *d* represents the hyperplane equation in *n*-dimensional space, $a_j, a_f \in \overrightarrow{\mathrm{A}_{\mathrm{j}} }$, where $\overrightarrow{\mathrm{A}_{\mathrm{j}}}= [a_1, a_2,\cdots , a_{n}\}$ is the attack vector.
- **Moving to new positions:** Moving to new positions of the golden eagles are mainly depends on the attack and cruise vectors. Therefore, the step vector of golden eagle *j* in iteration *t* is presented by the following equation:
  4 $$\begin{aligned} \Delta _{x_{j}}=\overrightarrow{\mathrm{r}_\mathrm{1}} p_a \frac{\overrightarrow{\mathrm{A}_{\mathrm{j}} }}{\bigg \Vert \overrightarrow{\mathrm{A}_{\mathrm{j}} }\bigg \Vert } + \overrightarrow{\mathrm{r}_\mathrm{2}} p_c \frac{\overrightarrow{\mathrm{C}_{\mathrm{j}} }}{\bigg \Vert \overrightarrow{\mathrm{C}_{\mathrm{j}} }\bigg \Vert } \end{aligned}$$
  where $p^t_a$ represents the attack coefficient at iteration *t* and $p^t_c$ represents the cruise coefficient at iteration *t* and control how the golden eagles are affected by cruise and attack. $\overrightarrow{\mathrm{r}_\mathrm{1}}$ and $\overrightarrow{\mathrm{r}_\mathrm{2}}$ are a random vectors.
  The new position of the golden eagle is then given by:
  5 $$\begin{aligned} x^{t+1}_j=x^t_j+ \Delta _{x_{j}}^t, \end{aligned}$$
  If the fitness function *j* provides better than the previous positions, then its memory will be updated with the new position.
- **Transition from exploration to exploitation:** GEO algorithm uses the attack coefficient $p_a$ and the cruise coefficient $p_c$ to switch from the state of exploration to the state of exploitation. $p_a$ and $p_c$ can be computed using the following linear expressions:
  6 $$\begin{aligned} p_a= & {} p_a^{0}+\frac{t}{T} |p_a^{T}-p_a^{0}|, \end{aligned}$$
  7 $$\begin{aligned} p_c= & {} p_c^{0}+\frac{t}{T} |p_c^{T}-p_c^{0}|, \end{aligned}$$
  where $p_a^{0}$ and $p_c^{0}$ are, respectively, the initial values for propensity to attack $p_a$ and for propensity to cruise $p_c$, *t* represents the current iteration, *T* is the maximum number of iterations, $p_a^{T}$ and $p_a^{T}$ are, respectively, the final values for propensity to attack $p_a$ and for propensity to cruise $p_c$.
  - **Optimized ANN model with golden eagle optimizer**
  The main inconvenient of ANN algorithm is that it can get stuck in local minimums easy and has a slow convergence rate. In recent years, researchers have shown that incorporating metaheuristics methods like GA^47^ and PSO^48–50^ in ANN, improves the performance of training process and the convergence rate significantly. However, GEO algorithm has been never used and investigated in training ANN. Training process involves identifying the corresponding set of influences that reduce the training error. Therefore, we proposed a new combined model that integrates GEO algorithm in the training of ANN to improve the prediction efficiency of ZnO NPs coated fabrics for thermophysiological properties. In this framework, ANN model is optimized by GEO algorithm in order to optimize the threshold and the weight, that significantly improves the prediction accuracy of the desired output. The flowchart of the proposed ANN-GEO model for comfort evaluation is presented in Fig. 3.

In ANN-GEO algorithm, at each iteration *t*, the golden eagle position $x^{j,t+1}$ is considered as the collection of weights. The MSE between the actual and predicted outputs is considered as the fitness function of ANN-GEO algorithm, where GEO seeks to minimize it during the ANN training process. Therefore, the fitness function is described by the following expression:

8 $$\begin{aligned} Minimize \{F\} = min \{\frac{1}{N}\Sigma _{j=1}^{N}{\Sigma _{i=1}^{n}{(y_{ji}-{\hat{y}}_{ji})^{2}}}\} \; \; \; where \; \; j=1,\cdots ,M\; and \; i=1,\cdots ,N. \end{aligned}$$

where, *M* is the the number of training samples and *N* is the number of output nodes.

The proposed ANN-GEO algorithm is mainly based on the following steps:

1. Initialization of the parameters of ANN-GEO algorithm: Golden eagle includes all weights and thresholds of ANN network i.e., connection weight for input and hidden layers, threshold for hidden layer, connection weight for hidden output layers, and threshold of output layer.
2. Initialization of memory, position, propensity to attack $p_a$ as well as propensity to cruise $p_c$ for every golden eagle: ANN-GEO initiates with random initialization of golden eagle positions where every golden eagle moves into the weighted search space.
3. Measurement of fitness function: Here, initial weights and bias are used to estimate initial training error.
4. Compute the attack vectors: Update the $p_a$ and $p_c$ values, and randomly select a prey. Then, computes attack vectors using Eq. (2).
5. Calculate the cruise and step vectors using Eqs. (3) and (4), respectively.
6. Compute the new position of the golden eagle using Eq. (5).
7. Estimate fitness function for new position, update $p_a$ and $p_c$ using Eqs. (6) and (7), respectively, and update memories of all golden eagles with best positions, and so on until the end of iterations.

### Robustness and sensitivity analysis

Prediction performance of thermophysiological comfort properties of ZnO NPs coated fabrics using ANN-GEO was estimated using statistical methods e.g., mean squared error (MSE), mean absolute error (MAE), root mean squared error (RMSE) and correlation coefficient (R), which are defined below.

9 $$\begin{aligned} MAE= & {} \frac{1}{m}\Sigma _{j=1}^{m}\vert {(y_j-{\hat{y}}_j)}\vert , \end{aligned}$$

10 $$\begin{aligned} MSE= & {} \frac{1}{m}\Sigma _{j=1}^{m}{(y_j-{\hat{y}}_j)^2}, \end{aligned}$$

11 $$\begin{aligned} RMSE= & {} \sqrt{\frac{1}{m}\Sigma _{j=1}^{m}{(y_j-{\hat{y}}_j)^2}}, \end{aligned}$$

12 $$\begin{aligned} R= & {} \frac{ \sum _{j=1}^{m}(y_j-{\bar{y}})(\hat{y_j}-\bar{{\hat{y}}}) }{ \sqrt{\sum _{j=1}^{m}(y_j-{\bar{y}})^2}\sqrt{\sum _{j=1}^{m}(\hat{y_j}-\bar{{\hat{y}}})^2}}. \end{aligned}$$

where $y_j$ and ${\hat{y}}$ are, respectively, the actual and predicted thermophysiological comfort properties. *m* represents the number of samples. ${\bar{y}}$ is the computed average of the actual properties and $\bar{{\hat{y}}}$ represents the computed average of the predicted properties.

The proposed model ANN-GEO was statistically tested using one-way ANOVA to evaluate its efficacy and durability comparing to others methods for the prediction of the thermophysiological comfort properties. ANOVA is an independent statistical approach to verify the statistical significance between inputs and outputs^51–53^. ANOVA uses *F* ratio to check the existence of any significant difference between the outputs.

## Results and discussion

### SEM and ICP-AES analysis

**Figure 4:**
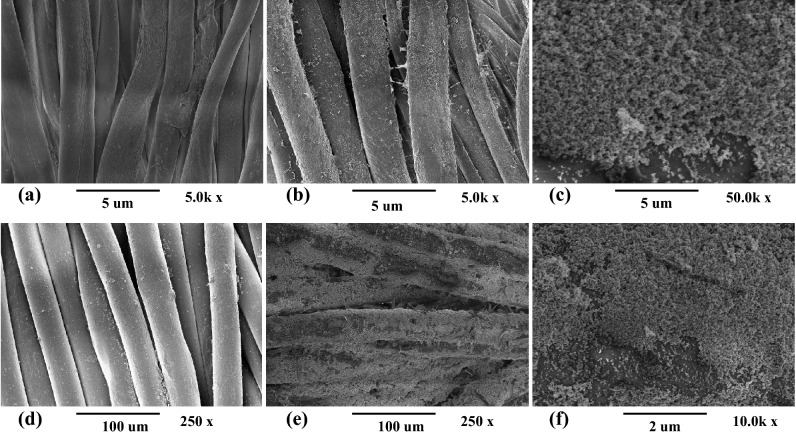
SEM images of (**a**) untreated cotton fabric, (**b**) ZnO coated cotton fabric, (**c**) ZnO coated cotton fabric with higher magnification, (**d**) untreated polyester fabric, (**e**) ZnO coated polyester fabric, (**f**) ZnO coated polyester fabric with higher magnification.

UHR-SEM was used for surface topography and morphology evaluation of treated and untreated samples as illustrated in Fig. 4. UHR-SEM images were taken at magnification 5k x and 50k x for cotton and at 250 x and 10k x for polyester respectively. A clean and smooth surface of untreated cotton and polyester can be observed in Fig. 4a,d respectively. A quasi spherical shape with homogeneous distribution of ZnO NPs was observed for both type of fabrics. In addition, ICP-AES analysis confirmed the presence of ZnO NPs on both fabrics. However, no traces were detected on untreated samples.

Comfort properties of ZnO NPs coated fabrics were determined through ANN under golden eagle optimizer (ANN-GEO). The obtained simulation results of ANN-GEO model were compared with standard ANN model, optimized ANN model with particle swarm optimization (ANN-PSO), optimized ANN model with genetic algorithm (ANN-GA) and optimized ANN model with crow search algorithm (ANN-CSA).

**Parameters setting of the optimized ANN models with metaheuristics algorithms** The proposed ANN model has three-layers i.e., an input layer, a hidden layer, and an output layer. After several trials, we found that the network provides highly accurate results with 9 hidden layer nodes, thus we considered that the number of hidden layer nodes for all proposed algorithms to be 9. The settings of training parameters of ANN, optimized models with GEO, PSO, CSA, and GA are introduced in Table 2.

**Table 2:** Parameters on ANN training network, optimized models with GEO, CSA, PSO, and GA.

Methods	Parameters	Settings
ANN	Training function	Trainlm
	Transfer function of hidden layer	Tansig
	Transfer function of output layer	Purelin
	Hidden node	9
	Input node	3
	Output node	5
	Performance goal	0.00001
	Learning rate	0.02
GEO	Population size	50
	Initial and final attack propensity	[0.5 2]
	Initial and final cruise propensity	[1 0.5]
	Lower and upper bound	[−0.7 0.7]
	Number of iterations	1000
PSO	Population size	50
	Inertia weight	1
	Cognitive factor C1	1.5
	Social factor C2	2
	Random values: r1, r2	[0,1]
	Number of iterations	1000
GA	Population size	50
	Crossover probability	0.4
	Variation probability	0.5
	Crossover method	Float
	Selection method	Roulette method
	Mutation method	Float
	Number of iterations	1000
CSA	Population size	50
	Awareness probability	0.1
	Flight length	2
	Number of iterations	1000

**Figure 5:**
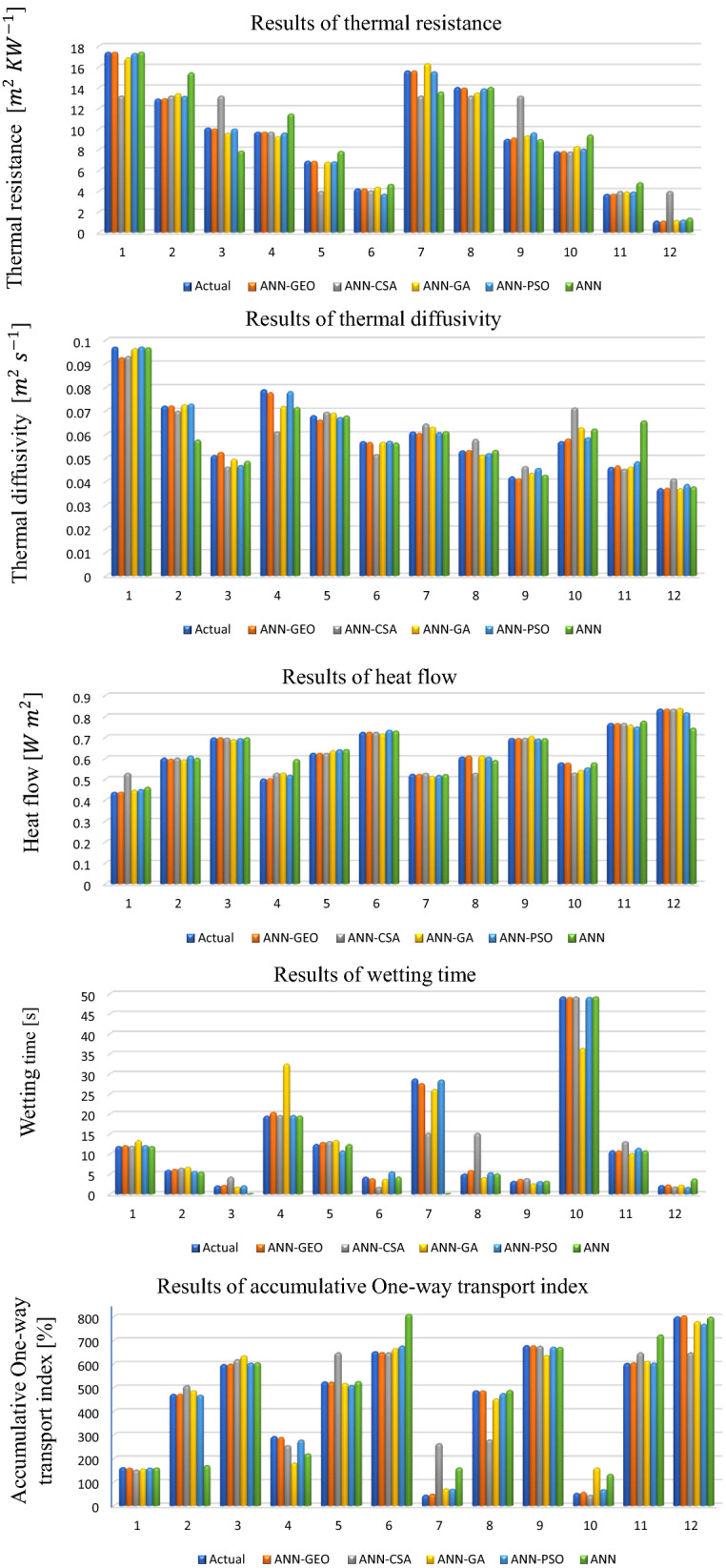
Simulation results for the prediction of thermophysiological comfort properties NPs coated fabrics, where thermal resistance (first row), thermal diffusivity (second row), heat flow (third row), wetting time (fourth row), and accumulative One-way transport index (last row) using ANN-GEO, ANN-CSA, ANN-PSO, ANN-GA and ANN.

**Comparison of ANN-GEO with currently used ANN-metaheuristics** The predicted values of comfort properties of ZnO NPs coated fabrics under standard ANN, ANN-GEO, ANN-PSO, ANN-GA and ANN-CSA are presented in Fig. 5, where predicted results for thermal resistance, thermal diffusivity, heat flow, wetting time and accumulative One-way transport index are presented from first row to fifth row respectively. We performed several trials with different number of populations (i.e., number of crows, number of swarm, etc) in each proposed algorithm in order to confirm a fair comparison between all applied algorithms. Then, we selected the best results with higher accuracy and lower errors for the prediction of comfort properties of ZnO NPs coated fabrics in each algorithm. For the stochastic nature of ANN-GEO, ANN-CSA, ANN-PSO, ANN-GA and ANN models, the prediction procedure of every property is repeated 1000 times and an average of 1000 times prediction is taken.

**Figure 6:**
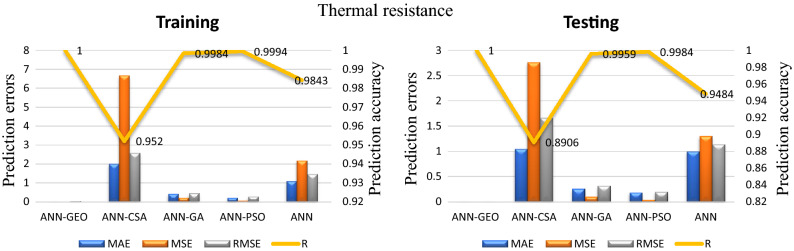
MAE, MSE, RMSE errors, and coefficient of correlation R between predicted and actual values of the thermal resistance using ANN-GEO, ANN-CSA, ANN-PSO, ANN-GA and ANN.

**Figure 7:**
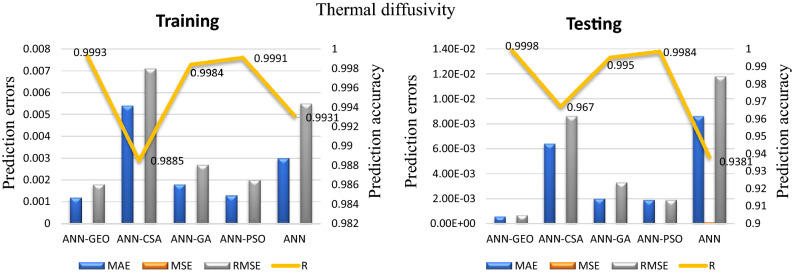
MAE, MSE, RMSE errors, and coefficient of correlation R between predicted and actual values of the thermal diffusivity using ANN-GEO, ANN-CSA, ANN-PSO, ANN-GA and ANN.

**Figure 8:**
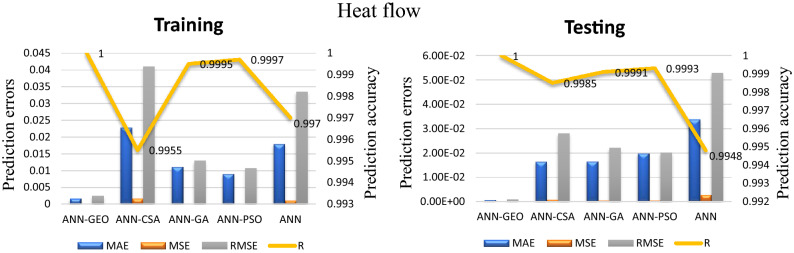
MAE, MSE, RMSE errors, and coefficient of correlation R between predicted and actual values of the heat flow using ANN-GEO, ANN-CSA, ANN-PSO, ANN-GA and ANN.

**Figure 9:**
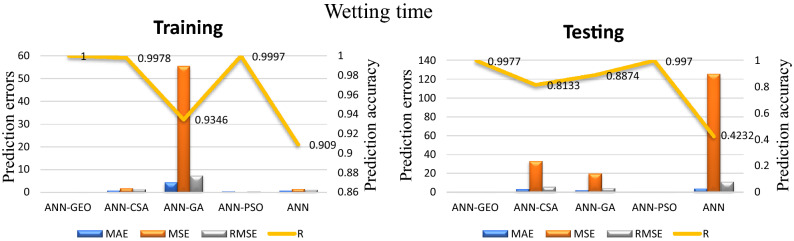
MAE, MSE, RMSE errors, and coefficient of correlation R between predicted and actual values of the wetting time using ANN-GEO, ANN-CSA, ANN-PSO, ANN-GA and ANN.

**Figure 10:**
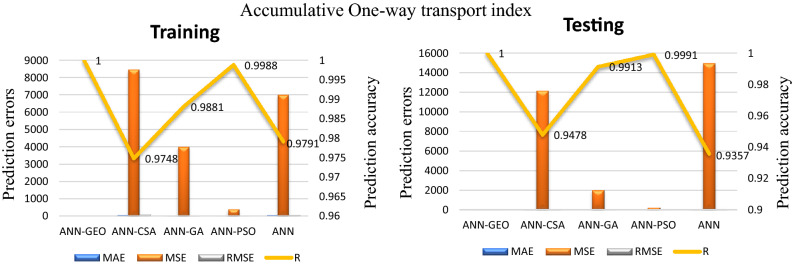
MAE, MSE, RMSE errors, and coefficient of correlation R between predicted and actual values of the accumulative One-way transport index using ANN-GEO, ANN-CSA, ANN-PSO, ANN-GA and ANN.

The values of prediction errors MAE, MSE, RMSE as well as the coefficient of correlation R between predicted and actual values for all evaluated comfort properties using ANN-GEO, ANN-GA, ANN-PSO, ANN-CSA and ANN are shown in Fig. 6 for thermal resistance, Fig. 7 for thermal diffusivity, Fig. 8 for heat flow, Fig. 9 for wetting time and Fig. 10 for accumulative One-way transport index respectively. We observed that ANN-GEO has lower prediction errors and higher prediction accuracy according to the coefficient of correlation ($R\approx 1$) for all evaluated outputs. The proposed ANN-GEO model significantly outperformed ANN-GA, ANN-PSO, ANN-CSA and standard ANN in both training and testing processes. Another observation found that ANN-PSO provides very good prediction accuracy than ANN-CSA, ANN-GA and standard ANN for comfort properties. However, ANN-CSA prediction results have the lower accuracy with higher prediction error.

The average time of each run for thermal resistance in GEO algorithm is 0.232 s, in PSO is 0.316 s, in GA is 0.322 s and in CSA is 0.689 s. The average time of each run for thermal diffusivity in GEO algorithm is 0.187 s, in PSO is 0.247 s, in GA is 0.352 s and in CSA is 0.478 s. The average time of each run for heat flow in GEO algorithm is 0.278 s, in PSO is 0.388 s, in GA is 0.421 s and in CSA is 0.762 s. The average time of each run for wetting time in GEO algorithm is 0.178 s, in PSO is 0.265 s, in GA is 0.369 s and in CSA is 0.698 s. The average time of each run for accumulative One-way transport index in GEO algorithm is 0.262 s, in PSO is 0.371 s, in GA is 0.578 s and in CSA is 0.829 s. It is clearly shown that convergence time with GEO is faster than other methods for all predicted properties, that strengthen the accuracy and effectiveness of the proposed ANN-GEO model.

In addition, we applied the conventional MLR method for performance evaluation of ANN-GEO model. The results obtained by MLR are as follows: for thermal resistance MAE=0.7595, MSE=0.739, RMSE=0.8597 and R=0.9676; for thermal diffusivity MAE=0.0075, MSE=0.93787, RMSE=0.0097 and R=0.8007; for heat flow MAE=0.0235, MSE=0.0912, RMSE=0.0302 and R=0.9621; for wetting time MAE=5.6642, MSE=56.3745, RMSE=7.5083 and R=0.8261; and for accumulative One-way transport index MAE=69.1001, MSE=6.23e+03, RMSE=78.9613 and R=0.9447. We observed that the results obtained by ANN-GEO model outperformed MLR for all outputs. The accuracy and the effectiveness of proposed ANN-GEO model is revealed by the obtained results.

**Figure 11:**
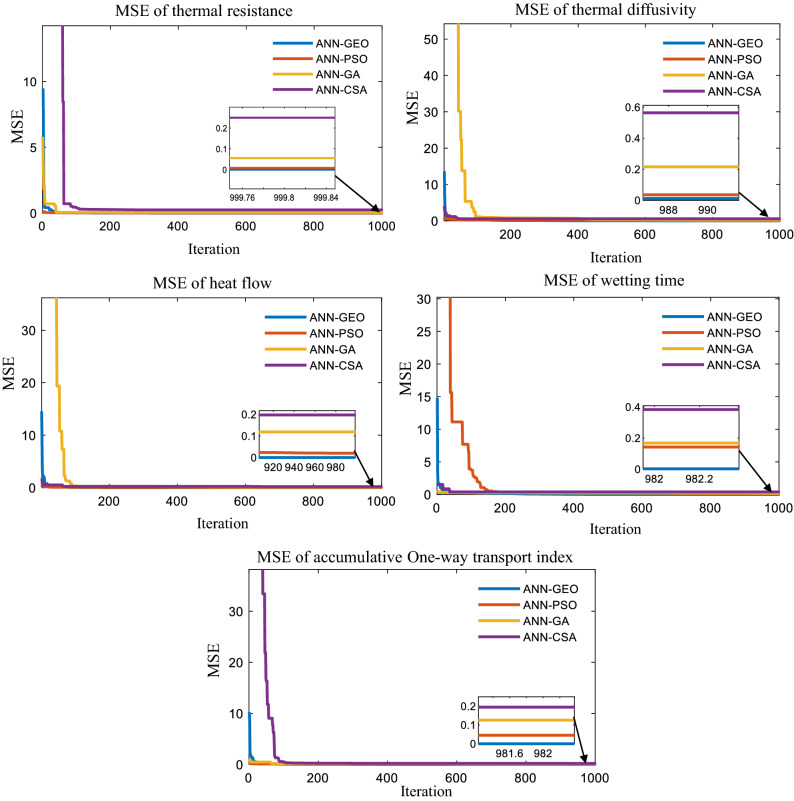
Performance MSE convergence characteristics for ANN-GEO, ANN-CSA, ANN-PSO and ANN-GA.

For all used optimized models, the performance MSE convergence characteristics have been shown in Fig. 11, where ANN-GEO is in blue, ANN-PSO is in red, ANN-CSA is in red, and ANN-GA is in mauve. We observed that the proposed MLP-GEO model provided the best results with lower MSE values as compared to ANN-PSO, ANN-CSA and ANN-GA for all thermophysiological properties.

**Robustness assessment** One-way ANOVA was performed to evaluate the effectiveness of predicted results using ANN-GEO, ANN-PSO, ANN-GA, ANN-CSA, ANN, MLR and experiment data. ANOVA is a statistical method aims to determine the correlation between variables and predicted results of ZnO coated fabrics. The results of each comfort property obtained by ANN-GEO, ANN-CSA, ANN-PSO, ANN-GA, ANN, MLR and experimental are illustrated in Table 3. It is observed that ANN-GEO model was more significant as compared to other models as it provide minimum $p-$value.

**Table 3:** Analysis report of the predicted values using ANN, ANN-CSA, ANN-PSO, ANN-GA, and ANN-GEO as well as the experimental values for the thermophysiological comfort properties NPs coated fabrics.

Thermophysiological comfort properties	Methods	*p*-value	F-value
Thermal resistance	ANN-GEO	3.22e-07	137.17
	ANN-PSO	8.05e-07	108.55
	ANN-GA	2.28e-06	83.01
	ANN-CSA	0.00016	26.45
	ANN	0.000029	42.52
	MLR	2.67e-6	79.7
	Experimental	2.67e-06	79.66
Thermal diffusivity	ANN-GEO	0.0057	9.18
	ANN-PSO	0.01517	6.53
	ANN-GA	0.0259	5.34
	ANN-CSA	0.0663	3.57
	ANN	0.03174	4.924
	MLR	0.0342	4.72
	Experimental	0.03417	4.77
Heat flow	ANN-GEO	2.91e-06	77.87
	ANN-PSO	5.74e-06	65.27
	ANN-GA	0.000059	35.05
	ANN-CSA	0.0003	21.61
	ANN	0.00026	23.14
	MLR	0.0000728	33.2
	Experimental	0.00007	33.21
Wetting time	ANN-GEO	0.01447	6.65
	ANN-PSO	0.022	5.619
	ANN-GA	0.027	5.20
	ANN-CSA	0.0306	4.99
	ANN	0.030	4.99
	MLR	0.0276	5.21
	Experimental	0.027	5.20
Accumulative One-way transport index	ANN-GEO	0.000024	44.68
	ANN-PSO	0.00011	28.99
	ANN-GA	0.00027	22.87
	ANN-CSA	0.0030	11.27
	ANN	0.00021	24.78
	MLR	0.0003	22.1
	Experimental	0.0003	22.13

## Conclusions

In this work, ANN-GEO is introduced as a novel model to enhance the prediction accuracy. ANN-GEO model is a combination of metaheuristics algorithms where GEO is employed for the first time to ameliorate the training process of ANN. ANN-GEO is used for the prediction of thermophysiological comfort of ZnO coated fabrics. The obtained results demonstrated that ANN-GEO model exhibits an efficient prediction accuracy ($$R>99\% $$) over standard ANN, ANN-PSO, ANN-CSA, ANN-GA and conventional MLR. Moreover, ANN-GEO showed lower error in terms of MAE, MSE and RMSE. ANN-GEO showed more statistical significant ($$p< 0.01147 $$) than other models. The findings of this study reveal that ANN-GEO can efficaciously be used for prediction as well as for classification of nanocomposites.
